# Integrated Molecular Docking with Network Pharmacology to Reveal the Molecular Mechanism of Simiao Powder in the Treatment of Acute Gouty Arthritis

**DOI:** 10.1155/2021/5570968

**Published:** 2021-04-27

**Authors:** Yihua Fan, Wei Liu, Yue Jin, Xu Hou, Xuewu Zhang, Hudan Pan, Hang Lu, Xiaojing Guo

**Affiliations:** ^1^First Teaching Hospital of Tianjin University of Traditional Chinese Medicine, Tianjin 300193, China; ^2^National Clinical Research Center for Chinese Medicine Acupuncture and Moxibustion, Tianjin 300381, China; ^3^Department of Endocrinology and Metabolic Diseases, Shandong Provincial Hospital Affiliated to Shandong First Medical University, Jinan 250021, Shandong Province, China; ^4^Department of Rheumatology, People Hospital of Beijing University, Bejing 100044, China; ^5^Dr. Neher's Biophysics Laboratory for Innovative Drug Discovery, State Key Laboratory of Quality Research in Chinese Medicine, Macau University of Science and Technology, Macao 999078, China

## Abstract

**Background:**

The incidence of gout has been rapidly increasing in recent years with the changing of diet. At present, modern medications used in the clinical treatment of gout showed several side effects, such as gastrointestinal damage and the increased risk of cardiovascular disease. The traditional Chinese prescription Simiao Powder (SMP) has a long history in the treatment of acute gouty arthritis (AGA) and has a good curative effect. However, the mechanism and target of its therapeutic effects are still not completely understood.

**Methods:**

Potential active compounds (PACs) and targets of SMP were found in the TCMSP database, and the disease target genes related to AGA were obtained by searching CTD, DisGeNET, DrugBank, GeneCards, TTD, OMIM, and PharmGKB disease databases with “acute gouty arthritis” and “Arthritis, Gouty” as keywords, respectively. The network of “Traditional Chinese medicine (TCM)-PACs-potential targets of acute gouty arthritis” was constructed with the Cytoscape 3.7.2 software, and the target genes of acute gouty arthritis were intersected with genes regulated by active compounds of SMP. The resultant common gene targets were input into Cytoscape 3.7.2 software, and the BisoGenet plug-in was used to construct a PPI network. The GO functional enrichment analysis and KEGG pathway enrichment analysis of the intersecting target proteins were performed using R software and corresponding program packages. The molecular docking verification was carried out between the potentially active compounds of SMP and the core target at the same time.

**Results:**

40 active components and 203 targets were identified, of which 95 targets were common targets for the drugs and diseases. GO function enrichment analysis revealed that SMP regulated several biological processes, such as response to lipopolysaccharide and oxidative stress, RNA polymerase II transcription regulator complex, protein kinase complex, and other cellular and molecular processes, including DNA-binding transcription factor binding. Results of KEGG pathway analysis showed that SMP was associated with AGA-related pathways such as interleukin-17 (IL-17), tumor necrosis factor (TNF), p53, and hypoxia-inducible factor 1 (HIF-1) signaling pathways. The results of molecular docking showed that active compounds in SMP exhibited strong binding to five core protein receptors (TP53, FN1, ESR1, CDK2, and HSPA5).

**Conclusions:**

Active components of SMP, such as quercetin, kaempferol, wogonin, baicalein, beta-sitosterol, and rutaecarpine, showed therapeutic effects on AGA. These compounds were strongly associated with core target proteins (such as TP53, FN1, ESR1, CDK2, and HSPA5). This study reveals that IL-17, TNF, p53, and HIF-1 signaling pathways mediate the therapeutic effects of SMP on AGA. These findings expand our understanding of the mechanism of SMP in the treatment of AGA.

## 1. Introduction

Gouty arthritis (GA) is characterized by acute onset of single joint redness, swelling, heat, and pain at night or in the morning. The pain progressively worsens, and in severe cases, patients often suffer from cutting pain, which often reaches a peak within 24 hours [1]. The number of gout patients has increased drastically due to dietary changes and high intake of high-purine foods and beer [2]. According to the gout epidemic survey in Hong Kong, the prevalence of gout increased from 1.56% in 2006 to −2.92% in 2016 [3]. In the United States, the prevalence of gout was about 3.9% in 2016, with an estimated 9.2 million gout patients [4]. Acute gouty arthritis (AGA) is the first symptom of gout. Severe pain can reduce the quality of life of patients. Recurrent acute arthritis can destroy joints and damage internal organs [5]. Gout treatment guidelines issued in 2012 by the American College of Rheumatology (ACR) recommend nonsteroidal anti-inflammatory drugs (NSAIDs) or oral colchicine for acute attacks of gout [6]. It has been reported that NSAIDs are harmful to the gastrointestinal tract, liver, and kidney, central nervous system, etc. [7]. Colchicine is an anti-inflammatory drug commonly used in the treatment of acute gout. However, when taken in large doses, it easily causes gastrointestinal tract discomfort, liver and kidney damage, or heart disease. Meanwhile, it may lead to poisoning in elderly patients [8]. Therefore, safer and more effective drugs for the treatment of AGA are urgently needed.

Simiao Powder (SMP) is a well-known prescription used in ancient China for the treatment of joint pain [9]. This prescription originated from *Chengfang Biandu* in the Qing Dynasty (1904 A.D.). SMP is composed of *Rhizoma Atractylodis* (Cang Zhu), *Cortex Phellodendri* (Huang Bo), *Radix Vladimiriae* (Niu Xi), and *Semen Coicis* (Yi Yi Ren), and it could clear heat, dry dampness, and promote blood circulation to invigorate the tendons. In the gouty arthritis model, Lin et al. revealed that SMP treatment could effectively relieve joint symptoms and improve laboratory indexes (e.g., serum uric acid content or inflammatory cytokines IL-1b, IL-9, IFN-g, and so on). Moreover, the anti-inflammatory effect of SMP was stronger than febuxostat [10]. A systematic review and meta-analysis demonstrated that SMP possesses anti-inflammatory, analgesic, and uric acid-lowering effects and hence can treat AGA. Jiawei reported that SMP is superior to other anti-inflammatory drugs or combined uric acid-lowering drugs. Compared to modern medications, SMP is associated with fewer adverse events [11]. However, the mechanism and the therapeutic target of SMP in the management of AGA are vague and are the main limiting factors for its widespread clinical application [11].

The entire human body is a complex network, and the occurrence and progression of a disease are the results of the comprehensive action of multiple factors. Single-targeted therapy cannot meet the needs of multitarget and multipathway treatment of diseases. The network pharmacology concept put forward by Hopkins in 2007 [12] provides a “multiway” and “multitarget” approach to drug analysis. It is similar to the thoughts of TCM compound prescription, as well as the characteristics of the overall TCM compatibility and comprehensive and multipathway treatment of diseases.

**Figure 1:**
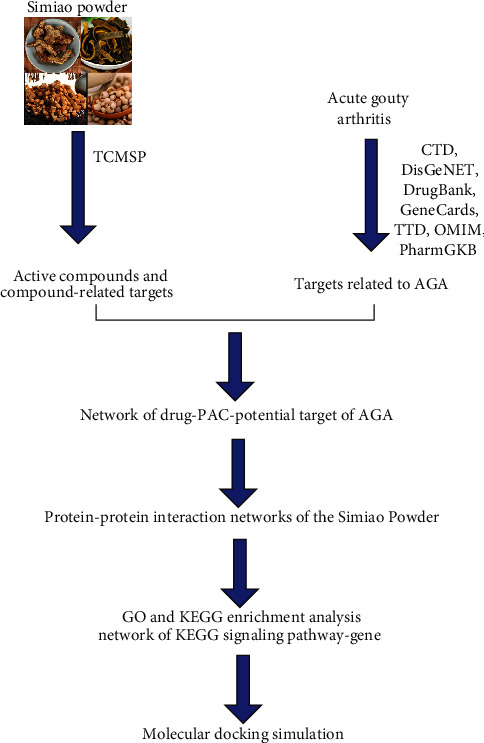
Flow diagram of the study.

The present study employed network pharmacology and molecular docking techniques to establish the active components of SMP in AGA treatment and predicted the potential target and drug action pathways of SMP. Collectively, we aimed to explore the potential mechanism of action of SMP and provide a reference for pharmacological research and clinical application of this prescription. The specific flow chart is shown in Figure 1.

## 2. Methods

### 2.1. Screening of Drug Components and SMP Targets

*Rhizoma Atractylodis* (Cang Zhu), *Cortex Phellodendri* (Huang Bo), *Radix Vladimiriae* (Niu Xi), and *Semen Coicis* (Yi Yi Ren), SMP components, were acquired from the traditional Chinese medicine systems pharmacology database and analysis platform (TCMSP, http://tcmspw.com/tcmsp.php), capturing all the potential active components of drugs in SMP. We screened the active compounds in SMP following the standards of oral bioavailability (OB) ≥30% and drug-likeness (DL) ≥0.18. Then, a search was undertaken for corresponding targets of active compounds on the TCMSP platform. The identified drug targets were input into the UniProt database (https://www.uniprot.org/). To obtain the abbreviation of the human gene for each target, we set the protein type as “Homo sapiens.”

### 2.2. Search and Screening of AGA-Related Genes

The search for target genes related to AGA was achieved using the terms “acute gouty arthritis” and “Arthritis, Gouty” in the Human Gene Database (GeneCards, https://www.genecards.org/), Online Mendelian Inheritance in Man (OMIM, https://www.omim.org/), the Pharmacogenomics Knowledge Base database (PharmGKB, https://www.pharmgkb.org/), DrugBank database (https://www.drugbank.ca/), DisGeNET database (https://www.disgenet.org/), the Therapeutic Target Database (TTD, http://db.idrblab.net/ttd/), and Comparative Toxicogenomics Database (CTD, http://ctdbase.org/). The results of the above databases were integrated to obtain the target genes of AGA after deleting the repeated genes.

### 2.3. Potential SMP Targets in the Treatment of AGA

We intersected the targets regulated by active compounds in SMP with the disease target genes of AGA. After that, common targets were obtained. The R 4.0.2 software and its Venn diagram package were employed to generate a Venn diagram of the SMP-AGA-related target.

### 2.4. Network Construction of Potential Active Compound-Potential Target

The potential targets and active compounds corresponding to the potential targets of SMP in AGA treatment were introduced into Cytoscape 3.7.2 software. Then, we constructed the network of “TCM-active compound-potential targets” and conducted the visualization display.

### 2.5. Construction of the Protein-Protein Interaction (PPI) Network and Screening of Core Targets

The intersection target was imported into Cytoscape 3.7.2 software. The PPI network was constructed via its plug-in BisoGenet with the following data sources: DIP, BIOGRID, HPRD, BIND, MINT, and INTACT. The plug-in CytoNCA was employed for topology analysis. Then, the degree centrality (DC) betweenness centrality (BC) of each node was calculated. A larger DC and BC of the node at the protein location implied that the protein was more important in the constructed PPI network [13]. Because we screened out various important target proteins, other protein interaction parameters, such as closeness centrality (CC) and eigenvector centrality (EC), were further applied to screen core target proteins.

### 2.6. Gene Ontology (GO) Functional Enrichment and the Kyoto Encyclopedia of Genes and Genomes (KEGG) Pathway Enrichment Analyses

The ID of the intersection target was obtained using R4.2.0 software and its “org.Hs.eg.db” package. Then, the “clusterProfiler” and other program packages were adopted for GO functional enrichment analysis of the target. A histogram was constructed after screening out the first 10 functional categories of biological process (BP), cellular component (CC), and molecular function (MF). The target proteins were subjected to the KEGG enrichment analysis, whereby the first 30 KEGG pathways were selected to construct the histogram. Information of the first 20 KEGG pathways was introduced into Cytoscape 3.7.2 to draw the KEGG pathway-gene network map. After that, we selected a signal pathway closely related to GA for visual display.

### 2.7. SMP Potential Active Compounds and Protein Molecular Docking

The 2D structure of the potential compound was retrieved from PubChem (https://pubchem.ncbi.nlm.nih.gov/). For small molecules, their energy was minimized using ChemBio3D 2014 software. The 2D structure was converted into a 3D structure. We downloaded the 3D structure of the core target protein from the Protein Data Bank (PDB) database (http://www.rcsb.org). Using the Pymol software, the target protein receptor molecules were processed, including dehydration and removal of ligand small molecules. The target protein receptor molecule was hydrogenated using Autodock Tools 4.2.6 software. The center coordinate and size of the box were set based on the position of the active site of the protein molecule and the area where it potentially acted on the ligand small molecule. Molecular docking was achieved using AutoDock Vina, whereby lower binding energy depicted a better affinity between the receptor and the ligand. The binding energy ≤0 kcal/mol indicated that the compound could bind and interact with the target, whereas the binding energy <−5 kcal/mol demonstrated a very strong binding force. The docking results with the best binding force between each core target protein and active compound were simultaneously presented in 2D images and 3D structures.

## 3. Results

### 3.1. Target Screening of SMP and AGA

**Figure 2:**
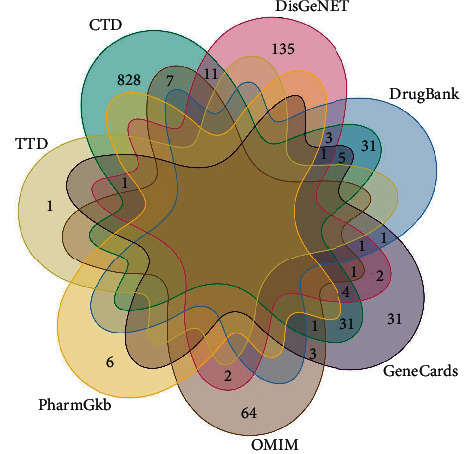
Venn diagram of disease targets.

In total, 403 compounds of SMP were obtained from the TCMSP database, including 49 from *Rhizoma Atractylodis* (Cang Zhu), 140 from *Cortex Phellodendri* (Huang Bo), 176 from *Radix Vladimiriae* (Niu Xi), and 38 from *Semen Coicis* (Yi Yi Ren). Besides, 52 active compounds were screened according to OB ≥ 30% and DL ≥ 0.18, including 4 from *Rhizoma Atractylodis* (Cang Zhu), 25 from *Cortex Phellodendri* (Huang Bo), 17 from *Radix Vladimiriae* (Niu Xi), and 6 from *Semen Coicis* (Yi Yi Ren). After eliminating repeated active compounds, 40 active compounds were obtained. From the TCMSP platform, 916 targets of the 40 active compounds were obtained, including 60 from *Rhizoma Atractylodis* (Cang Zhu), 430 from *Cortex Phellodendri* (Huang Bo), 384 from *Radix Vladimiriae* (Niu Xi), and 42 from *Semen Coicis* (Yi Yi Ren). After eliminating repeated targets, 203 targets and their abbreviations were obtained. Information of active compounds and the number of action targets of TCM in SMP are outlined in Table 1. Through a search in GeneCards, OMIM, PharmGKB, DrugBank, DisGeNET, TTD, and CTD databases, 1,204 target genes associated with AGA were obtained (Figure 2).

### 3.2. Potential Targets and Corresponding Active Compounds of SMP in the Treatment of AGA

**Figure 3:**
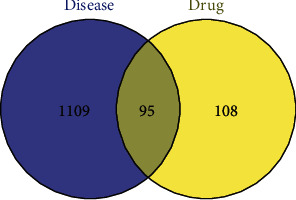
Venn diagram of drug targets and disease targets.

The drug-disease intersection target (the potential target of SMP in AGA treatment) was obtained by intersecting the 203 drug targets with 1204 disease targets using R software, with 95 potential targets (Figure 3). Notably, 33 active compounds corresponded to the 95 potential targets. They were the active compounds of SMP in AGA treatment.

### 3.3. Construction of TCM-PACs-Potential Target of AGA Network

**Figure 4:**
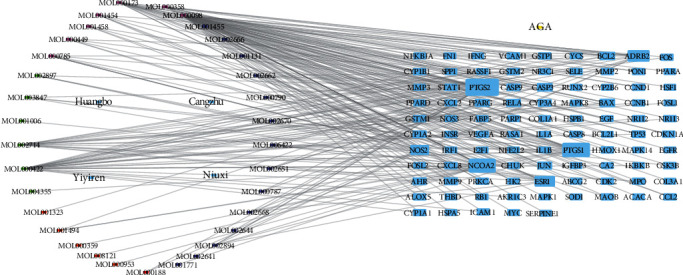
A network of the drug-PAC-potential target of AGA. The left circle represents the active ingredients. Different colors distinguish drug sources of each ingredient; blue represents the active ingredient from Cortex Phellodendri (Huang Bo), yellow represents the active ingredient from Rhizoma Atracylodis (Cang Zhu), orange represents the active ingredient from Semen Coicis (Yi Yi Ren), green represents the active ingredient from Radix Vladimiriae (Niu Xi), and purple represents the common ingredient of various drugs. The square node on the right denotes the target.

We drew and analyzed the potential targets of SMP in the treatment of AGA using Cytoscape 3.7.2 network drawing software. Then, a PAC-potential target network diagram of SMP for AGA treatment was constructed (Figure 4). The network diagram comprised 133 nodes (including 95 target genes, 33 drug active components, 4 TCM, and 1 disease name) and 250 edges. We further calculated the degree of the compound in the figure. Notably, a higher degree implied that the compound played a more critical role in the network. The top six active compounds included MOL000098-quercetin, MOL000422-kaempferol, MOL000173-wogonin, MOL002714-baicalein, MOL000358beta-sitosterol, and MOL002662-rutaecarpine, with 79, 30, 21, 18, 12, and 7 as the corresponding target numbers, respectively.

### 3.4. Construction of PPI Network, Topological Analysis, and Determination of Core Targets

**Figure 5:**
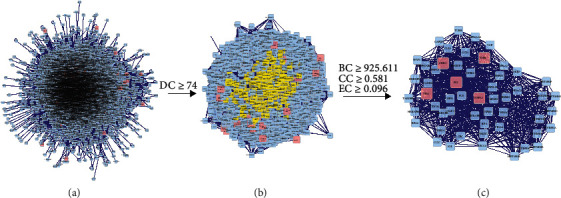
Protein-protein interaction network of the Simiao Powder.

After obtaining the potential target genes, they were imported into Cytoscape 3.7.2 software. We then drew the PPI network diagram using the Bisogent plug-in and obtained 5,264 nodes and 133,011 connections (Figure 5(a)). For the first topology analysis, DC ≥ 74 and 982 nodes and 43,726 connections were obtained (Figure 5(b)). The second topology analysis was conducted with BC ≥ 925.611 (average), CC ≥ 0.581 (average), and EC ≥ 0.096 (average) to identify the crucial target genes; eventually, 54 nodes and 829 lines were obtained. Target proteins with the top five degree values were selected as the core target proteins in the protein network (Figure 5(c)), where TP53 protein, FN1 protein, ESR1 protein, CDK2 protein, and HSPA5 protein corresponded to 946 edges, 781 edges, 1,762 edges, 687 edges, and 445 edges, respectively.

### 3.5. GO Functional Enrichment and KEGG Pathway Enrichment Analyses

**Figure 6:**
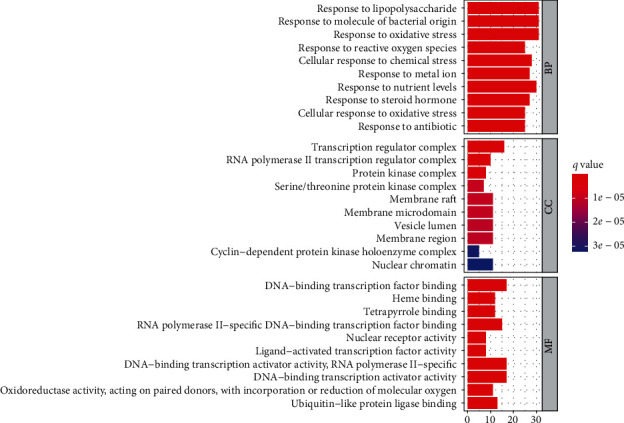
GO functional enrichment analysis.GO analysis of putative targets. The *y*-axis demonstrates the top 10 significantly enriched BP, CC, and MF categories, whereas the *x*-axis displays the number of enrichment genes of these terms (*P* < 0.05). The color denotes the different *P* value range, and the redder it is, the more significant enrichment.

For GO analysis, BP, CC, and MF were included, and 1,938 items were obtained via BP enrichment analysis (including response to lipopolysaccharide, oxidative stress, reactive oxygen species (ROS), etc.); 36 items via CC enrichment analysis (including RNA polymerase II transcription regulator complex, protein kinase complex, and membrane raft); 145 items via MF enrichment analysis (including DNA-binding transcription factor binding, heme binding, and RNA polymerase II-specific DNA-binding transcription factor binding). To draw the GO function histogram of SMP in AGA treatment, we selected the top 10 BP, CC, and MF results at *P* < 0.05 (Figure 6).

**Figure 7:**
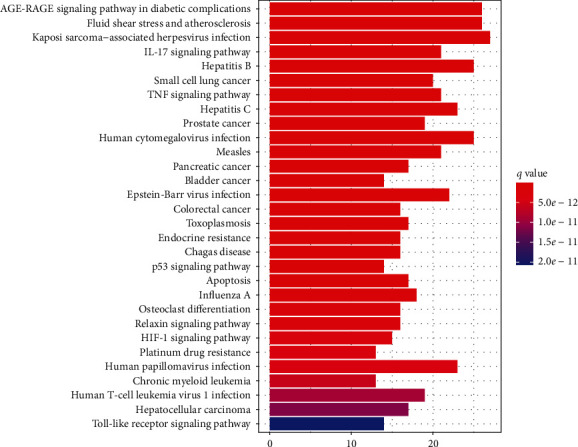
KEGG pathway enrichment analysis. *Note.* KEGG enrichment analysis of putative targets. The *y*-axis outlines the top 30 significantly enriched KEGG pathways, whereas the *x*-axis displays the number of enrichment genes of these terms (*P* < 0.05). The color denotes the different *P* value range; the redder it is, the more significant enrichment.

**Figure 8:**
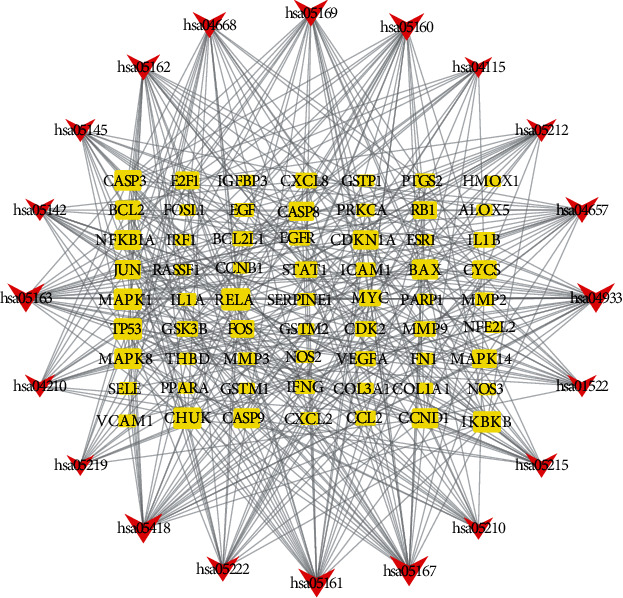
A network of KEGG signaling pathway-gene. Red triangles denote pathways, whereas yellow square nodes denote genes. The larger the shape of the node is, the more genes or pathways are connected to it.

**Figure 9:**
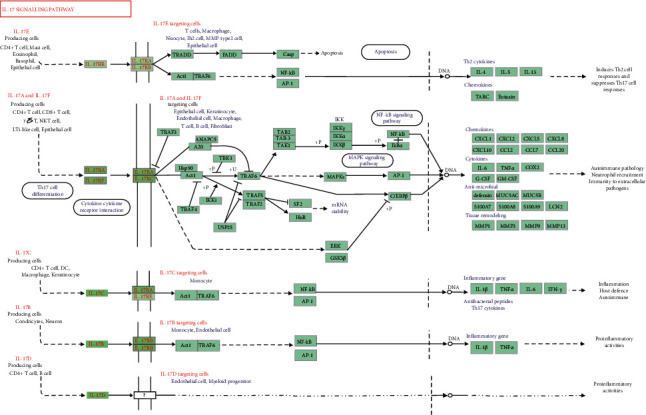
The IL-17 signaling pathway. The green nodes in the road map denote the genes existing in our SMP network.

To further elucidate the pathways regulated by the therapeutic target genes, we did a KEGG pathway analysis. Results revealed that these target genes were distributed in 155 pathways. To construct a histogram, the pathways with the top 30 enriched genes were selected (Figure 7). GA-related pathways were mainly associated with IL-17, TNF, p53, and HIF-1 signaling pathways. We also analyzed the association of the top 20 pathways with their corresponding target genes by constructing a KEGG pathway-gene network diagram (Figure 8). There were 83 nodes (including 20 pathways and 63 target genes) and 403 lines. Based on the number of regulatory pathways, the top five genes were RELA, TP53, MAPK1, IKBKB, and CHUK with 16, 15, 15, 15, and 15 regulatory pathways, respectively. A strong relationship existed between GA and inflammation (the IL-17 pathway is closely related to inflammation). Therefore, we constructed a diagram to demonstrate the association of the regulatory target of SMP with the IL-17 pathway (Figure 9).

### 3.6. Molecular Docking

**Figure 10:**
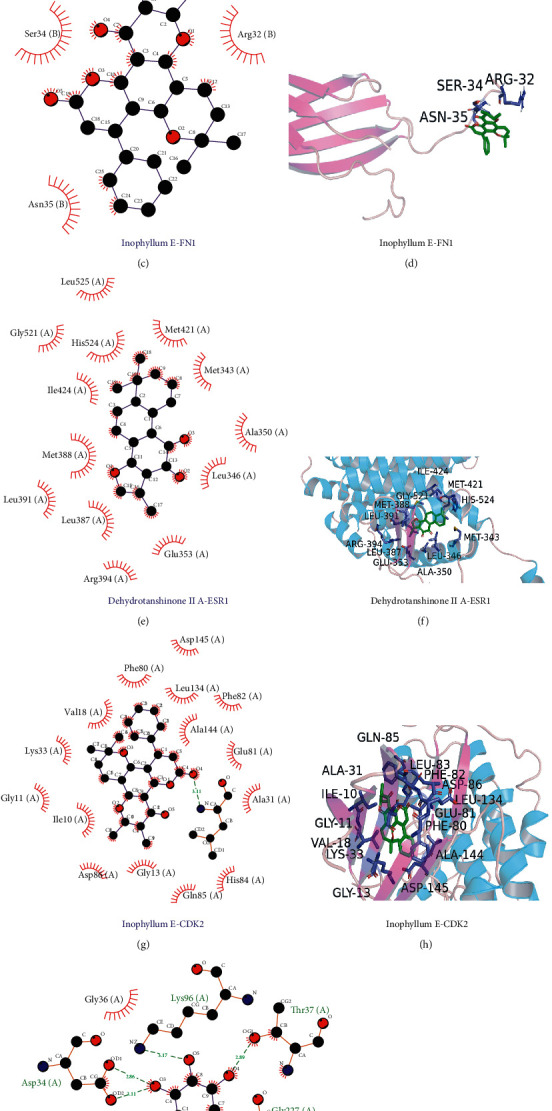
A diagram for the five core targets molecular docking of SMP gouty arthritis treatment.

Using the AutoDock Vina software, PACs in the network map of “TCM-PAC-potential targets of AGA” were docked with five core target proteins (TP53, FN1, ESR1, CDK2, and HSPA5) in PPI. A higher binding activity between the compound and the target protein receptor implied lower binding energy. Results demonstrated that the docking of 33 active compounds with the 5 core target protein receptors was less than 0 kcal/mol (a majority was less than −5 kcal/mol) (Table 2). The molecular docking results of the compounds with the highest binding energy corresponding to the five targets are displayed in 2D images and 3D structures (Figure 10).

## 4. Discussion

The repeated attacks and protracted course of acute gouty arthritis can lead to joint swelling, deformity, and destruction of bone and joint cartilage. Tophi can be formed in many parts of the body, and it could seriously invade joints, kidneys, and even aorta and heart valves in severe cases [14–16]. For the treatment of gout, anti-inflammatory analgesics are mainly used in the acute stage. After the pain of patients is completely relieved, the use of uric acid-lowering drugs to correct the serum uric acid level is adopted [7, 17, 18]. Studies have found that although febuxostat and allopurinol can effectively reduce blood uric acid levels, they can also increase the risk of cardiovascular disease [19]. Traditional Chinese medicine compounds could function in anti-inflammatory analgesics and lowering uric acid at the same time, which has certain advantages compared with anti-inflammatory analgesics alone [20–22]. Besides, its relatively fewer adverse reactions and lower price have attracted more and more scholars' attention.

According to Figure 4, the key active components of SMP in the treatment of GA were quercetin, kaempferol, wogonin, baicalein, beta-sitosterol, and rutaecarpine. Quercetin is the active compound of *Cortex Phellodendri* (Huang Bo) and *Radix Vladimiriae* (Niu Xi), kaempferol is the active compound of *Radix Vladimiriae* (Niu Xi), wogonin is the active compound of *Rhizoma Atractylodis* (Cang Zhu) and *Radix Vladimiriae* (Niu Xi), baicalein is the active compound of *Radix Vladimiriae* (Niu Xi), beta-sitosterol is the active compound of *Cortex Phellodendri* (Huang Bo) and *Radix Vladimiriae* (Niu Xi), and rutaecarpine is the active compound of *Cortex Phellodendri* (Huang Bo). Among them, quercetin and wogonin are the common active components of two traditional Chinese medicines, which is similar to the compatibility and synergism of TCM compounds. Considering *Semen Coicis* (Yi Yi Ren) as an indispensable TCM in this prescription, the active ingredient stigmasterol, characterized by a great degree value in Figure 4, was established as one of the crucial active compounds.

These PACs in SMP have a certain curative effect on inhibiting inflammation and lowering blood uric acid level [23]. Among them, quercetin, baicalein, wogonin, and rutaecarpine showed strong anti-inflammatory effects [19–21]. Baicalein could inhibit the activity of NF-*κ*B and some inflammatory factors, such as monocyte chemotactic protein-1 (MCP-1), interleukins, tumor necrosis factor, and cellular adhesion molecules. In addition, it also clears the reactive oxygen species (ROS) to show anti-inflammatory and antioxidant effects [24]. Moreover, wogonin, a flavonoid from medicinal plants, has shown various biological activities. It potentially inhibits the production of inflammatory mediators by macrophages and lymphocytes [25]. Inhibition of NF-*κ*B and mitogen-activated protein kinase (MAPK) pathways in rutaecarpine-derivative R3 inhibits the NLRP3 inflammasome activation, which consequently lowers the expression of proinflammatory cytokine IL-1*β* [26, 27]. The pathogenesis of GA is highly associated with the inflammatory cascade induced by the activation of the inflammatory bodies of NLRP3 [2]. Thus, the inhibitory effect of rutaecarpine on the inflammatory body activation of NLRP3 may influence GA treatment. Besides, quercetin and kaempferol may significantly elevate the total antioxidant capacity and inhibit the xanthine oxidase (XOD) effect in hyperuricemia rats [28]. In another study, inhibition of XOD activity could reduce the serum uric acid level and the formation of ROS, consequently reducing the deposition of gout urate crystals [29]. Additional studies found that quercetin could reduce serum uric acid levels in hyperuricemia mice and impede renal insufficiency. This was achieved via the regulation of renal organic ion transport protein and uromodulin, and demonstrated fewer side effects than allopurinol [30, 31]. Beta-sitosterol (SIT), the main compound among various phytosterols, can reduce the expression of chemokines and inflammatory cytokines. It then regulates various biological functions, including anti-inflammatory, analgesic, immunomodulatory, and antimicrobial functions [32, 33]. Also, stigmasterol can inhibit the inflammation induced by sodium urate crystals and XOD activity in mice, a phenomenon that validates its antigout effect [34].

Herein, to obtain the core target proteins of SMP in the treatment of GA disease, the PPI network of intersecting target proteins was constructed. Also, we performed two topological screens on the SMP target for GA treatment based on DC, BC, CC, and EC. TP53, FN1, ESR1, CDK2, and HSPA5 were the five core target proteins. Based on the existing reports, TP53, also known as p53, contributes to transcriptional regulation of the cell cycle, DNA repair, cell survival, and cell metabolism [35]. Inhibition of p53 exerts a substantial effect on promoting the senescence of IL-1*β*-induced chondrocytes [36]. The potential role of TP53 in the regulation of inflammation has been described, whereby it significantly inhibits the production of proinflammatory factor IL-6 [37]. The overexpression of FN1 can activate TGF-Akt/PI3K/Akt signal pathway to promote cell viability and differentiation capacity [38]. Cyclin-dependent kinase (CDK) is the main regulator of cell division. It can be potentially triggered by different cyclins at different cell cycle stages [39], and it can regulate the secretion of inflammatory cytokines in macrophages [40] and inhibit CDK2. In consequence, a reduction in chemotaxis of primary neutrophils outlines the role of CDK2 in regulating inflammation [39]. Estrogen receptor 1 (ESR1) is a subtype of human ESR, expressed in chondrocytes, stromal cells, and osteoblasts [41]. In particular, ESR can induce the transcription of related target genes to promote the proliferation and differentiation of tissue cells as they bind to estrogen [42]. Heat shock protein family A member 5 (HSPA5) gene can also participate in the inflammatory process of various types of osteoarthritis as it encodes the binding immunoglobulin protein (BiP) of the endoplasmic reticulum Hsp70 family [43].

Following the KEGG signal pathway analysis, the mechanisms of SMP in the treatment of AGA were mainly anti-inflammation and protection of the cartilage. The critical inflammatory signaling pathways included TNF and IL-17, whereas the apoptosis signaling pathways included p53 and HIF.

Current pieces of research on the mechanism of gout have reported that the phagocytosis of MSU crystal triggers the change of inflammatory cell state of synovial cells in GA pathogenesis [14]. For instance, the deposition of MSU crystals promotes the local release of TNF-*α* and IL-1*β*, which causes persistent gout episodes [44]. IL-17 also stimulates macrophages and monocytes to produce proinflammatory factors, including IL-1*β*, TNF-*α*, and IFN-*γ* (Figure 9). As a powerful proinflammatory cytokine, IL-17 can increase serum IL-17 levels 8 hours following the acute attack of gout [45]. Moreover, in several arthritic animal models, the inhibition of IL-17 expression was found to limit inflammation and reduce joint erosion [46]. The TNF signaling pathway has also proved to be a crucial inflammatory signal pathway. Signal transduction is mediated by TNF receptor 1 (TNFR1) and receptor-2 (TNFR2). TNFR2 is, in most cases, expressed in immune cells. It binds to TNF-*α* and TNF-*β* to regulate immune response [47]. TNF-*α* is a proinflammatory cytokine produced by different cell types, such as macrophages, lymphocytes, and fibroblasts, and reflects inflammation, infection, and other environmental stresses [48]. Previous reports showed that inhibition of TNF-*α* expression in synovial cells during AGA could effectively inhibit local inflammation [49, 50].

The physiological dose of soluble uric acid exerts a specific protective effect on cartilage [51]. However, following repeated attacks by GA, the articular cartilage edge is damaged, the articular surface becomes irregular, the joint space becomes narrow, and MSU deposition occurs [52]. A study found that reduced articular cartilage matrix secretion in patients with GA was associated with chondrocyte apoptosis [53]. p53 can regulate cell differentiation and senescence. The inhibition of p53 expression potentially improves senescence and affects the destruction of IL-1*β*-induced chondrocytes [36]. Compared to the inflammatory signaling pathway, the HIF-1 signaling pathway is slightly less in gout studies. As a transcription factor, activation of HIF-1*α* expression in synovial cells may be induced during hypoxia. Synovial hyperplasia follows the repeated infiltration of inflammatory cells in the joint. On the other hand, hypoxia is associated with immune cell infiltration and synovial hyperplasia. Therefore, the upregulation of synovium-derived HIF-1*α* occurs in the pathogenesis of rheumatoid arthritis (RA) and osteoarthritis (OA), which are hypoxia-associated diseases [54, 55]. HIF-1*α* exerts a regulatory role in the function of immune cells. Activated LPS-induced macrophages can also express HIF-1*α*, which is crucial in glycolysis and the induction of proinflammation [56]. The therapeutic effect of SMP on AGA may be derived through the regulation of the inflammatory signal pathway, p53 apoptosis signal, and HIF-1signaling pathway. And other top-ranked signaling pathways, such as the AGE-RAGE signaling pathway in diabetic complications, fluid shear stress, and atherosclerosis, etc., still lack their gout-related studies, which are likely to be the future direction of gout mechanism research.

According to the space matching and energy matching relationship between molecules, the core target protein of SMP in AGA treatment was docked with active compounds. This provides a reference for the subsequent research and development of targeted drugs. The 2D image of molecular docking depicts the binding mode between the core target protein and the compound as well as the interaction with the surrounding amino acid residues. Hydrophobic interaction best describes the main association of target protein TP53, FN1, and ESR1 with Inophyllum E. The target protein CDK2 residue Leu83 (A) forms a hydrogen bond with Inophyllum E, whereas the residues Asp34 (A), Lys96 (A), Thr37 (A), and Gly227 (A) of the target protein HSPA5 form five hydrogen bonds with baicalein. The main forces between ligand and protein are hydrophobic force and hydrogen bond, which both are chemical bonds with strong binding force. According to Table 2, key components of SMP have strong binding activity with core target proteins, such as TP53, FN1, ESR1, CDK2, and HSPA5, suggesting that SMP may play its pharmacological role by regulating these key targets. However, there are still some shortcomings in this study, and the conclusions need to be further verified by in vivo, in vitro, and clinical trials.

## 5. Conclusion

The present study systematically expounds the core compounds and molecular action mechanism of SMP in the treatment of AGA. The core compounds of SMP, including quercetin, kaempferol, wogonin, baicalein, and beta-sitosterol, were screened via network pharmacology, whereas the core targets of AGA including TP53, FN1, ESR1, CDK2, and HSPA5 were screened out via PPI. These core compounds are characterized by stable binding activity to the core targets. Also, SMP could regulate signal pathways related to AGA disease, for example, TNF, IL-17, p53, and HIF signaling pathways. These findings suggest the potential synergistic role of SMP in treating AGA via multiple components, multitargets, multiple biological functions, and multiple signal pathways.

## Figures and Tables

**Table 1:** Active compounds and their number of targets of SMP.

No.	Source	ID	Compound	OB	DL	Number
1	Huangbo, Niuxi	MOL000098	quercetin	46.43	0.28	138
2	Niuxi	MOL000422	kaempferol	41.88	0.24	55
3	Cangzhu, Niuxi	MOL000173	wogonin	30.68	0.23	41
4	Niuxi	MOL002714	baicalein	33.52	0.21	33
5	Huangbo	MOL000790	Isocorypalmine	35.77	0.59	31
6	Huangbo, Niuxi	MOL000358	beta-sitosterol	36.91	0.75	28
7	Huangbo	MOL001455	(S) -Canadine	53.83	0.77	28
8	Huangbo, Niuxi, Yiyiren	MOL000449	Stigmasterol	43.83	0.76	27
9	Huangbo	MOL002670	Cavidine	35.64	0.81	24
10	Huangbo	MOL000787	Fumarine	59.26	0.83	22
11	Huangbo	MOL002651	Dehydrotanshinone II A	43.76	0.4	18
12	Cangzhu	MOL000188	3*β*-acetoxyatractylone	40.57	0.22	17
13	Huangbo, Niuxi	MOL000785	palmatine	64.6	0.65	16
14	Huangbo	MOL002662	rutaecarpine	40.3	0.6	14
15	Huangbo, Niuxi	MOL001454	berberine	36.86	0.78	14
16	Huangbo	MOL006422	thalifendine	44.41	0.73	12
17	Huangbo	MOL002894	berberrubine	35.74	0.73	11
18	Huangbo	MOL002644	Phellopterin	40.19	0.28	9
19	Niuxi	MOL002897	piberberine	43.09	0.78	9
20	Huangbo, Niuxi	MOL001458	coptisine	30.67	0.86	8
21	Huangbo	MOL001131	phellamurin_qt	56.6	0.39	7
22	Niuxi	MOL003847	Inophyllum E	38.81	0.85	6
23	Huangbo	MOL002668	Worenine	45.83	0.87	6
24	Yiyiren	MOL001323	Sitosterol alpha1	43.28	0.78	5
25	Huangbo	MOL002666	Chelerythrine	34.18	0.78	5
26	Yiyiren	MOL000953	CLR	37.87	0.68	3
27	Niuxi	MOL004355	Spinasterol	42.98	0.76	3
28	Huangbo	MOL002663	Skimmianin	40.14	0.2	3
29	Yiyiren	MOL000359	sitosterol	36.91	0.75	3
30	Niuxi	MOL001006	poriferasta-7	42.98	0.76	3
31	Huangbo	MOL002641	Phellavin_qt	35.86	0.44	3
32	Yiyiren	MOL001494	Mandenol	42	0.19	3
33	Huangbo	MOL001771	poriferast-5-en-3beta-ol	36.91	0.75	2
34	Huangbo	MOL000622	Magnograndiolide	63.71	0.19	2
35	Cangzhu	MOL000184	NSC63551	39.25	0.76	1
36	Huangbo, Niuxi	MOL002643	delta 7-stigmastenol	37.42	0.75	1
37	Huangbo	MOL005438	campesterol	37.58	0.71	1
38	Cangzhu, Niuxi	MOL000085	beta-daucosterol_qt	36.91	0.75	1
39	Yiyiren	MOL008121	2-Monoolein	34.23	0.29	1
40	Niuxi	MOL012461	28-norolean-17-en-3-ol	35.93	0.78	1

**Table 2:** The affinity of the putative compounds with core targets.

Compound	Affinity -TP53 (kcal/mol)	Affinity-FN1 (kcal/mol)	Affinity-ESR1 (kcal/mol)	Affinity-CDK2 (kcal/mol)	Affinity -HSPA5 (kcal/mol)
Dehydrotanshinone II A	−7.6	−4.0	−8.7	−11.5	−8.3
3*β*-acetoxyatractylone	−6.8	−3.3	−8.7	−8.6	−6.9
baicalein	−6.6	−4.0	−8.4	−8.7	−9.7
kaempferol	−6.4	−3.9	−8.3	−8.4	−8.8
wogonin	−6.9	−3.9	−8.3	−8.7	−7.9
quercetin	−6.5	−3.9	−8.1	−8.8	−8.2
Phellopterin	−6.6	−3.4	−7.9	−8.5	−7.0
phellamurin_qt	−7.4	−3.8	−7.2	−9.4	−7.8
Mandenol	−4.3	−1.6	−7.1	−6.4	−4.2
rutaecarpine	−8.3	−4.2	−7.0	−10.5	−9.6
2-Monoolein	−4.9	−2.1	−6.6	−6.5	−4.7
Spinasterol	−7.9	−3.8	−6.5	−9.1	−8.3
beta-sitosterol	−7.3	−3.4	−6.5	−10.6	−7.6
Phellavin_qt	−7.1	−3.7	−6.2	−9.5	−7.7
CLR	−7.0	−3.2	−6.1	−9.9	−8.1
poriferast-5-en-3beta-ol	−7.6	−3.1	−6.1	−9.9	−7.0
sitosterol	−6.8	−3.1	−6.0	−10.2	−7.7
Stigmasterol	−7.8	−3.9	−5.9	−9.8	−8.1
Sitosterol alpha1	−7.2	−3.6	−5.6	−9.8	−8.4
Fumarine	−7.4	−3.9	−5.5	−9.5	−8.9
Chelerythrine	−7.6	−3.6	−5.5	−10.4	−8.2
Worenine	−7.7	−4.3	−5.4	−10.9	−9.0
poriferasta-7	−7.1	−3.5	−5.3	−10.4	−7.6
epiberberine	−6.8	−3.8	−5.1	−9.4	−8.6
Cavidine	−6.9	−3.9	−4.9	−8.7	−8.1
berberine	−7.3	−3.8	−4.8	−9.6	−8.0
thalifendine	−6.8	−4.0	−4.7	−9.6	−8.3
(S)-Canadine	−6.7	−3.8	−4.7	−9.1	−8.1
berberrubine	−6.9	−3.8	−4.7	−9.5	−7.6
Inophyllum E	−8.6	−4.4	−4.2	−11.7	−9.7
coptisine	−7.4	−4.0	−4.2	−10.1	−8.9
Isocorypalmine	−6.5	−3.9	−4	−8.7	−7.5
palmatine	−6.5	−3.6	−3.6	−8.8	−7.2

## Data Availability

The data for this study can be provided by the corresponding author (Wei Liu: fengshiliuwei@163.com).

## Abbreviations

SMP: Simiao Powder
AGA: Acute gouty arthritis
TCM: Traditional Chinese medicine
IL-17: Interleukin-17
TNF: Tumor necrosis factor
HIF-1: Hypoxia-inducible factor 1
GA: Gouty arthritis
ACR: American College of Rheumatology
NSAIDs: Nonsteroidal anti-inflammatory drugs
TCMSP: Traditional Chinese medicine systems pharmacology database and analysis platform
OB: Oral bioavailability
DL: Drug-likeness
OMIM: Online Mendelian Inheritance in Man
PharmGKB: Pharmacogenomics Knowledge Base database
TTD: Therapeutic Target Database
CTD: Comparative Toxicogenomics Database
PACs: Potential active compounds
PPI: Protein-protein interaction
DC: Degree centrality
BC: Betweenness centrality
CC: Closeness centrality
EC: Eigenvector centrality
GO: Gene ontology
KEGG: Kyoto Encyclopedia of Genes and Genomes
BP: Biological process
CC: Cellular component
MF: Molecular function
PDB: Protein Data Bank
ROS: Reactive oxygen species
MSU: Monosodium urate
NF-*κ*B: Nuclear factor-kappa B
MAPK: Mitogen-activated protein kinase
XOD: Xanthine oxidase
SIT: Beta-sitosterol
XO: Xanthine oxidase
CDK: Cyclin-dependent kinase
ESR1: Estrogen receptor 1
HSPA5: Heat shock protein family A (Hsp70) member 5
BiP: Binding immunoglobulin protein
TNFR1: TNF receptor 1
TNFR2: TNF receptor-2
RA: Rheumatoid arthritis
OA: Osteoarthritis.
